# The complete mitochondrial genome sequence of *Schizothorax griseus* (Cypriniformes: Cyprinidae)

**DOI:** 10.1080/23802359.2017.1375880

**Published:** 2017-09-11

**Authors:** Xue Wang, Sheng Zeng, FeiXiong Chen, ZhengYou Li

**Affiliations:** a Guizhou Fisheries Research Institute, Guiyang, Guizhou, P. R. China;; b Guizhou Academy of Agricultural Sciences, Guiyang, Guizhou, P. R. China

**Keywords:** *Schizothorax griseus*, mitochondrial genome, diversification, biogeography, evolutionary mechanisms

## Abstract

In this study, the complete mitochondrial genome (mtDNA) of *Schizothorax griseus* was sequenced. The mitogenome is 16,586 bp in length, composed of 13 protein-coding genes, two rRNA genes, 22 tRNA genes, and two non-coding regions. The phylogenetic relationship constructed by Bayesian inference (BI) method showed that *schizothorax* is not a monophyly, species in the same river express a more close relationship. The results can contribute to explore the high level of diversification and the taxonomic conflict caused by morphologic characters of *schizothorax*, also, it will be an evidence for the biogeography and evolutionary mechanisms of Schizothoracinae fishes.

Schizothoracinae fish is a divergent group which is evaluated from mountain areas of Central Asia, there are more than 90 species in China that are mainly distributed around the Qinghai-Tibetan Plateau regions (Chen and Cao 2000; Dai and Xiao 2011). *Schizothorax griseus* is distributed in upstreams of Wujiang river, Nanpanjiang river and Beipanjiang river (Chen and Cao 2000), it has been listed in ‘China Species Red List’ and ‘Red list of China’s Vertebrates’ as endangered (Wang and Xie 2009; Cao et al. 2016). The sample (GZ 20170628) caught from Nayong county, Guizhou province (N27°2′12.8″, E105°13′8.5″) was deposited in the collection of the Guizhou Fisheries Research Institute, Guiyang, China. Total genomic DNA was extracted from the pelvic-fin preserved in 95% alcohol using the Qiagen QIAamp tissue kit following the manufacturer’s protocol. We designed pairs of primers for PCR amplification and sequenced the complete mitochondrial genome.

After assembling and alignment, the complete mitogenome of *S. griseus* was determined to be 16,586 bp in length (Genbank accession no. MF 688995), containing 13 protein-coding genes (*cytb*, *ATP6*, *ATP8*, *COI-III*, *ND1-6*, and *ND4L*), two ribosomal RNA genes (12srRNA and 16srRNA), 22 transfer RNA (tRNA) genes, two non-coding regions: the control region (D-loop) and the origin of light-strand (rep-region: O_L_). Except *ND6* and eight tRNA genes (tRNA^Gln^, tRNA^Ala^, tRNA^Asn^, tRNA^Cys^, tRNA^Tyr^, tRNA^Ser^, tRNA^Glu^, and tRNA^Pro^), all these genes were encoded on the H-strand. The overall nucleotide base composition base was: 25.4% T; 27.1% C; 17.8% G and 29.7% A, showing a weak anti-G bias which was nearly the same in 13 protein-coding genes (17.7% G). All protein-coding genes shared typical start codon ATG except for *COI* beginning with GTG, only six protein-coding genes terminated with the complete stop codon, the remaining had the incomplete stop codon. There were six regions of gene overlap totalling 21 bp (from 1 to 7 bp) and 10 intergenic spacer regions totalling 30bp (from 1 to 12 bp). The D-Loop, with 937 bp in length, was located between tRNA^Pho^ and tRNA^Phe^, and the base composition reflected a higher A + T-rich (66.9%) than the overall average mtDNA (55.1%).

Here, we used Bayesian inference (BI) method to construct phylogenetic tree between *S. griseus* and 25 complete mitogenome sequences downloaded from GenBank, *Percocypris pingi, Ptychobarbus dipogon*, and *Aspiorhynchus laticeps* were chosen as outgroup (Figure 1). The results showed that *Schozothorax* did not cluster as a monophyletic group but reflect geographical associations with rivers, which is in accordance with recent researches (He and Chen 2006; Yang and Chen 2012). The outgroup *Percocypris pingi* (subfamily: Barbinae) has a more close relationship with species in genus *Schizothorax* and may contribute to origin of Schizothoracinae fishes for that we usually accepted Schizothoracinae was derived from Barbinae (Wu and Chen 1980; Cao et al. 1981). We hoped that the complete mitochondrial DNA sequence of *S. griseus* can contribute to explore the high level of diversification and the taxonomic conflict caused by morphologic characters of *Schizothorax*, also, we hoped the conclusion can be an evidence for the biogeography and evolutionary mechanisms of Schizothoracinae fishes.

**Figure 1.**
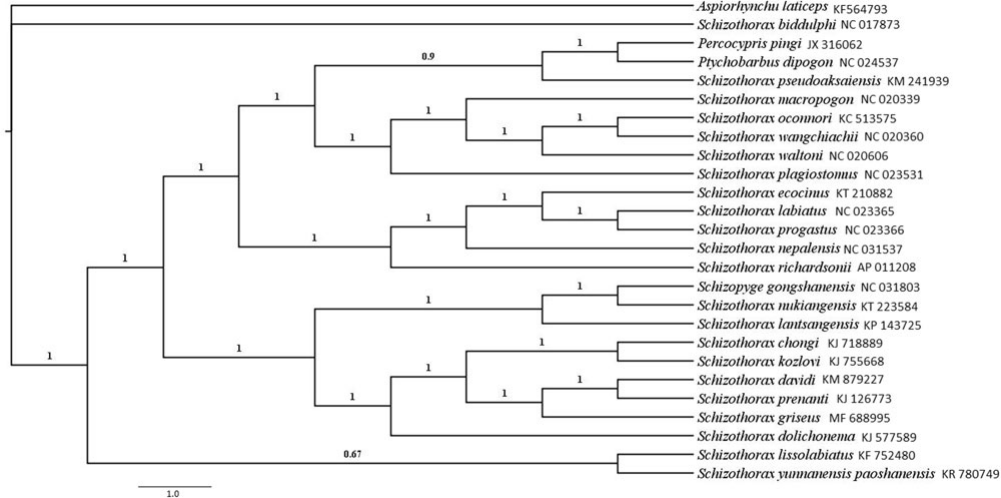
Phylogenetic relationships among the genus Schizothorax using Bayesian analysis based on complete mitochondrial genome. Numbers on the nodes are Bayesian posterior probability.
